# Using metabolic potential within the airway microbiome as predictors of clinical state in persons with cystic fibrosis

**DOI:** 10.3389/fmed.2022.1082125

**Published:** 2023-01-09

**Authors:** Gabriella Shumyatsky, Aszia Burrell, Hollis Chaney, Iman Sami, Anastassios C. Koumbourlis, Robert J. Freishtat, Keith A. Crandall, Edith T. Zemanick, Andrea Hahn

**Affiliations:** ^1^Jefferson Biotechnology Program, Thomas Jefferson University, Philadelphia, PA, United States; ^2^Center for Genetic Medicine Research, Children’s National Research Institute, Washington, DC, United States; ^3^Department of Pediatrics, George Washington University (GWU), Washington, DC, United States; ^4^Division of Pulmonary Medicine, Children’s National Hospital (CNH), Washington, DC, United States; ^5^Division of Emergency Medicine, CNH, Washington, DC, United States; ^6^Department of Biostatistics and Bioinformatics, Milken Institute School of Public Health, GWU, Washington, DC, United States; ^7^Department of Pediatrics, University of Colorado Anschutz Medical Campus, Aurora, CO, United States; ^8^Division of Infectious Diseases, CNH, Washington, DC, United States

**Keywords:** cystic fibrosis, lung disease, microbiome, bacterial gene, metabolic pathway

## Abstract

**Introduction:**

Pulmonary exacerbations (PEx) in persons with cystic fibrosis (CF) are primarily related to acute or chronic inflammation associated with bacterial lung infections, which may be caused by several bacteria that activate similar bacterial genes and produce similar by-products. The goal of our study was to perform a stratified functional analysis of bacterial genes at three distinct time points in the treatment of a PEx in order to determine the role that specific airway microbiome community members may play within each clinical state (i.e., PEx, end of antibiotic treatment, and follow-up). Our secondary goal was to compare the change between clinical states with the metabolic activity of specific airway microbiome community members.

**Methods:**

This was a prospective observational study of persons with CF treated with intravenous antibiotics for PEx between 2016 and 2020 at Children’s National Hospital. Demographic and clinical information as well as respiratory samples were collected at hospital admission for PEx, end of antibiotic treatment, and follow-up. Metagenomic sequencing was performed; MetaPhlAn3 and HUMANn3 were used to assign sequences to bacterial species and bacterial metabolic genes, respectively.

**Results:**

Twenty-two persons with CF, with a mean age of 14.5 (range 7–23) years, experienced 45 PEx during the study period. Two-hundred twenty-one bacterial species were identified in the respiratory samples from the study cohort. Ten bacterial species had differential gene abundance across changes in the clinical state including *Staphylococcus aureus*, *Streptococcus salivarius*, and *Veillonella atypica* (all padj < 0.01 and log2FoldChange > |2|). These corresponded to a differential abundance of bacterial genes, with *S. aureus* accounting for 81% of the genes more abundant in PEx and *S. salivarius* accounting for 83% of the genes more abundant in follow-up, all compared to the end of treatment. Lastly, 8,653 metabolic pathways were identified across samples, with again *S. aureus* and *S. salivarius* contributing to the differential abundance of pathways (106 in PEx vs. 66 in follow-up, respectively). *V. atypica* was associated with a single metabolic pathway (UDP-*N*-acetyl-D-glucosamine biosynthesis) increased in follow-up compared to PEx.

**Discussion:**

Taken together, these data suggest that the metabolic potential of bacterial species can provide more insight into changes across clinical states than the relative abundance of the bacteria alone.

## 1. Introduction

Cystic fibrosis (CF) is a genetic disease caused by the absence or dysfunction of the cystic fibrosis transmembrane conductance regulator (CFTR) protein (1). As a result, persons with CF commonly experience impaired pulmonary mucociliary clearance and subsequent chronic airway infections (2). Moreover, the lungs of CF patients are unable to properly clear the damaging byproducts of the inflammatory immune responses associated with chronic airway infections (3). When a response to infection causes an acute decrease in lung function or patient health (referred to as a pulmonary exacerbation, PEx), antibiotic intervention becomes necessary to regain pulmonary function (4). This cycle of chronic infection, inflammation, damage, treatment, and re-infection creates a negative feedback loop where the more damage the lungs sustain, the more likely another infection will occur. Additionally, many external genetic factors and genotypic variants of CF contribute to the disease phenotype of the CF individual that make it very difficult to create a universal treatment solely based on host genotype or phenotype (5). Beyond CFTR modulators and broad antibiotic treatments directed at common CF airway pathogens, insights may be gained in observing the variation in the CF airway microbiome across clinical states (6–9). More recent work also suggests that the metabolome could be used to predict or diagnose pulmonary exacerbation (10, 11).

The goal of our study was to perform a stratified functional analysis of bacterial genes associated with the clinical states of PEx hospitalization, end of antibiotic treatment, and follow-up in order to determine the role that specific airway microbiome community members may play within each clinical state. Our secondary goal was to compare the change between clinical states with the metabolic activity of specific airway microbiome community members. Our hypothesis is that differences in gene expression of specific airway community members is detectable between clinical states and correlates to differences in metabolic activity between clinical states. These findings will provide insight into the mechanisms behind changes in clinical state as well as airway microbiome community members’ contributions to these changes.

## 2. Materials and methods

### 2.1. Study design

This single-center prospective observational study enrolled persons with CF treated with intravenous (IV) antibiotics for PEx between 2016 and 2020 at Children’s National Hospital in Washington, DC. Study approval was obtained from the Institutional Review Board at Children’s National (Pro6781, 8 Dec 2015 and Pro10528, 31 Aug 2018). Written informed consent was obtained from study participants ≥18 years of age, with written parental consent from children <18 years of age. Assent was obtained for children 11–17 years of age. Respiratory samples and clinical data were collected at during three clinical states: the initiation of IV antibiotics for PEx, at the end of antibiotic treatment, and at their next follow-up visit, an adaptation of the BETR criteria (6, 12, 13). Study participants could re-enroll in the study if they had another exacerbation during the study period; as such, many study participants contributed respiratory samples for more than one PEx.

### 2.2. Respiratory sample collection and processing

Sputum, oropharyngeal (OP) swab, or bronchoalveolar lavage (BAL; all performed clinically) specimens were collected and processed with standard procedures previously described (12–14). Briefly, respiratory samples were collected in sterile containers and held at 4^°^C until processing. Microbiologic cultures were performed in the clinical laboratory. For future metagenomic sequencing, sputum and BAL specimens were mixed with equal volumes of Sputasol (Thermofisher) and sterile normal saline, followed by vortexing and heating in a 37^°^C bead bath to homogenize the sample. Homogenized samples and the Amies media from OP swabs were centrifuged at 12,000 *g* × 10 min to pellet the cells. Supernatants and cells were frozen separately at −80^°^C until DNA extraction.

### 2.3. Bacterial DNA extraction and metagenomic sequencing

Bacterial DNA extraction and metagenomic sequencing were performed with standard procedures as previously described (13, 15). Briefly, frozen cell pellets were thawed at room temperate and mixed with sterile phosphate buffered saline (PBS). The QIAamp DNA Microbiome Kit (Qiagen) was used following the manufacturer’s protocol to extract bacterial DNA. Qubit (Thermofisher Scientific) was used to measure DNA quantity and Bioanalyzer (Agilent) was used to assess DNA quality. Libraries were constructed using a Nextera XT Library Prep Kit (Illumina). A Mid-Output 2 × 150 cycle kit was used to sequence 20–30 libraries per run on a NextSeq 500 (Illumina). The respiratory samples were also sequenced with ZymoBiomics Microbial Community Standards (Zymo Research) as controls.

### 2.4. Bioinformatic analysis

Evaluation of sequences for quality and for sequence trimming was done using FastQC and Flexbar (16). Human sequences were filtered out using KneadData (17). MetaPhlAn3 was used to assign bacterial taxonomy (18), using the function “rel_ab_w_read_stats” to generate count tables. HUMAnN3 was used to analyze the sequence data of the samples for gene, pathway, and associated microbiome community member (18). MetaCycwas utilized for pathway collection (19). The function “humann_regroup” was used to assign Gene Ontology (GO) values from the original uniref90 gene output. Relative abundance and count tables were imported into R for subsequent analyses and figure generation. Rstudio packages used for analysis included *DESeq2* v.1.24.0 (20), *ggplot2* v3.2.0 (21), *phyloseq* v.1.28.0 (22), and *vegan* v.2.5-6 (23). Bacterial species observed, Shannon index, and the inverse Simpson’s index were calculated using the *specnumber* and *diversity* functions, respectively. Permutational analysis of variance (PERMANOVA) was also performed in Rstudio using the *adonis* function for analysis and portioning sums of squares using Bray-Curtis dissimilarities. Repeated patient samples were controlled for using the *strata* function. *DESeq2* was used to perform the differential abundance analysis between time points for bacterial taxonomy, bacterial genes, and bacterial metabolic pathways (20), utilizing a log fold change shrinkage for visualization and ranking (24). STATA/IC (v15.1) was used for statistical analysis to compare these measures across time points.

## 3. Results

### 3.1. Study cohort and clinical characteristics

Twenty-two persons who had a total of 45 PEx were included in the study (Table 1), with 13 of 22 study participants contributing respiratory samples for multiple PEx. The median age was 16.5 years (range 7–23). The majority were male (59%) and non-Hispanic White (55%). Sixty-eight percent had at least one copy of the F508del mutation of the CFTR gene. For 42% of the PEx, the study participant was receiving a CFTR modulator (ivacaftor *n* = 5, ivacaftor/lumacaftor *n* = 12, and tezacaftor/ivacaftor *n* = 2). At onset of the first PEx, the mean body mass index was 19.4 (SD 3.5), and the mean best percent predicted forced expiratory volume in one second (ppFEV1) in the prior 6 months was 81.4% (SD 25.9%). The most common bacteria identified by respiratory culture were *Pseudomonas aeruginosa* (47%), methicillin-resistant *Staphylococcus aureus* (20%), and methicillin-sensitive *Staphylococcus aureus* (16%). The most common antibiotics received for the PEx were tobramycin (62%), ceftazidime (32%), and meropenem (27%). The most common antibiotics directed against methicillin-resistant *Staphylococcus aureus* were vancomycin and trimethoprim-sulfamethoxazole (each 13%). The median antibiotic course for the PEx was 14 days. Using a paired *t*-test, we found a significant difference in ppFEV1 between PEx and end of treatment (65.1 vs. 80.3, *p* < 0.001, observations = 42 of 45 total PEx) and between end of treatment and follow-up (80.4 vs. 70.5, *p* < 0.001, observations = 41 of 45 total PEx).

**TABLE 1:** Study cohort demographics and clinical features.

Study participant characteristics	*N* = 22
Age at first PEx (mean years, SD)	14.6 (5.0)
Sex (*n*, % female)	9 (41%)
**Race/Ethnicity (*n*, %)**	
Non-Hispanic White	12 (54.5%)
Non-Hispanic Black	1 (4.5%)
Hispanic	9 (41%)
**CFTR genotype (*n*, %)**	
F508del homozygous	10 (45.5%)
F508del heterozygous	5 (22.7%)
Other	7 (31.8%)
**Pulmonary exacerbation characteristics**	***N* = 45**
Current culture results (*n*, %)*	
Methicillin-sensitive *Staphylococcus aureus*	7 (16%)
Methicillin-resistant *Staphylococcus aureus*	9 (20%)
*Streptococcus pyogenes*	1 (2%)
*Pseudomonas aeruginosa*	21 (47%)
*Achromobacter xylosoxidans*	1 (2%)
*Burkholderia cepacia*	3 (7%)
*Burkholderia multivorans*	1 (2%)
*Burkholderia gladioli*	3 (7%)
*Acinetobacter baumanii*	1 (2%)
*Pseudomonas putida*	1 (2%)
Unidentified gram-negative rod	1 (2%)
Antibiotics used (*n*, %)*	
Piperacillin-tazobactam	7 (16%)
Ceftazidime	16 (36%)
Cefepime	9 (20%)
Ceftaroline	1 (2%)
Meropenem	12 (27%)
Aztreonam	5 (11%)
Tobramycin	28 (62%)
Amikacin	2 (4%)
Ciprofloxacin	2 (4%)
Levofloxacin	4 (4%)
Vancomycin	6 (13%)
Linezolid	3 (7%)
Clindamycin	1 (2%)
Doxycycline	3 (7%)
Trimethoprim-sulfamethoxazole	6 (13%)
Total antibiotic days (mean, SD)	15.2 (4.9)

PEx, pulmonary exacerbation; SD, standard deviation; CFTR, cystic fibrosis transmembrane conductance regulator.

*Totals do not add to 100% as many cultures had multiple organisms and multiple antibiotics were used for each PEx.

### 3.2. Bacterial abundance and diversity identified by metagenomic sequencing

**FIGURE 1:**
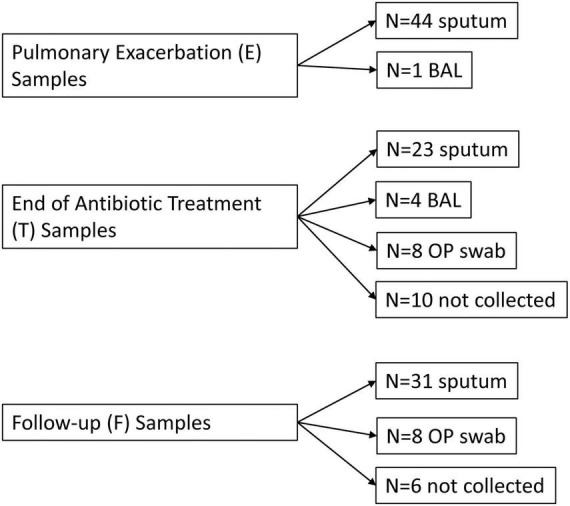
Flow diagram of respiratory samples by time point. E, pulmonary exacerbation; T, end of antibiotic treatment; F, follow up; BAL, bronchoalveolar lavage; OP, oropharyngeal.

All PEx respiratory samples were collected and sequenced (see Figure 1 for breakdown of sample type). Thirty-three end of treatment respiratory samples were collected and successfully sequenced (2 failed sequencing, 10 were not collected). Thirty-nine follow-up samples were collected and sequenced (6 were not collected). As a sensitivity analysis was previously performed of our sample collection and sequencing methods which showed good congruence between sample types (13), we did not adjust for sample type in our subsequent analyses. Five Zymo sequencing controls showed significant correlation with their expected community compositions (R 0.743–0.812, R^2^ 0.552–0.659, all *p* < 0.01). The clinical samples had an estimated number of reads from the clades in the range of 37.8 K to 7.7 million.

**FIGURE 2:**
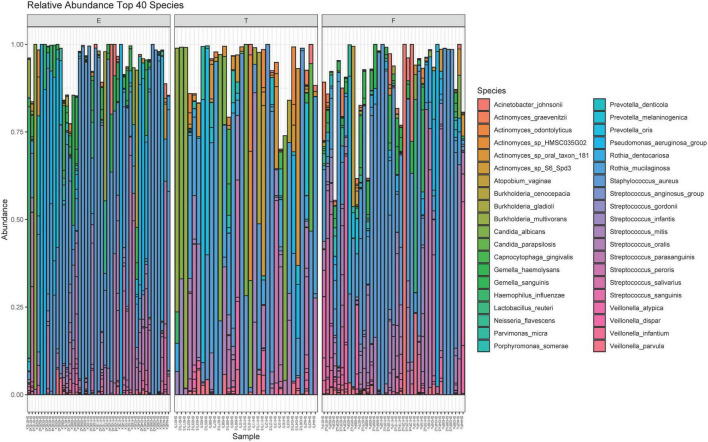
Relative abundance plot of the top 40 species observed. The 40 species with the highest contribution to the dataset are included here. The bar plot for each sample can approach a maximum relative abundance of 1.00. In cases where the bar is <1.00, it is because the remaining species contributing to that respiratory sample’s community are from the remaining 181 species observed in the dataset. E, pulmonary exacerbation; T, end of treatment; F, follow up.

**FIGURE 3:**
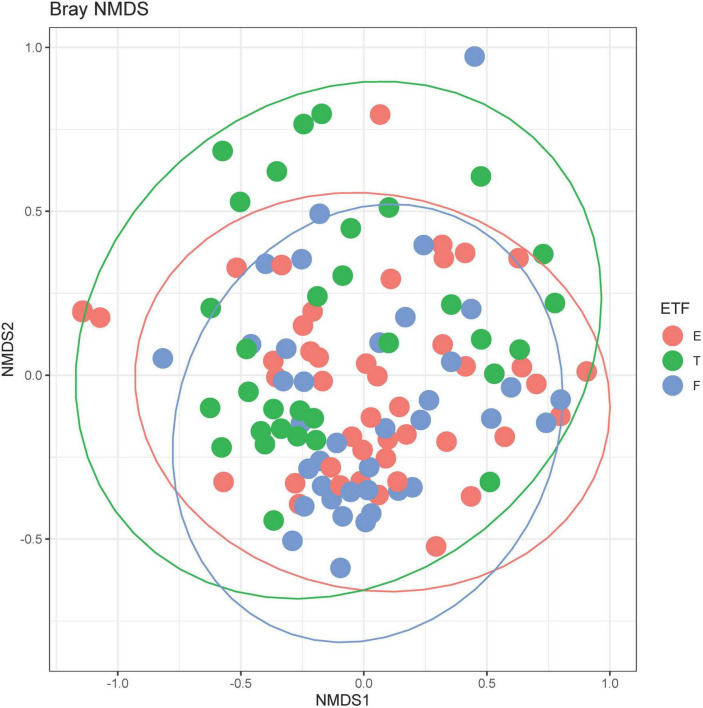
Bray-Curtis non-metric multidimensional scaling (NMDS) plot. E, pulmonary exacerbation; T, end of treatment; F, follow up. The ellipses represent the *t* distribution. The *adonis* test was significant at *p* = 0.001, controlling for repeated patient samples using the *strata* function.

A total of 211 bacterial species were identified across all respiratory samples, with a range of 1–86 species observed per sample (Figure 2). We also compared alpha diversity between time points using a paired *t*-test (Table 2). We did not find significant differences between PEx and end of treatment but did between end of treatment and follow up. We also found a significant difference in beta diversity as measured by the Bray-Curtis index, which appeared to be driven by the shift in the end of treatment samples (Figure 3). Additionally, we evaluated the differential abundance of bacterial species after first normalizing the count data to relative abundance (25). Only *Gemella haemolysans* was increased in PEx compared to end of treatment (log2 fold change 4.7, padj < 0.001). *Gemella haemolysans* (log2 fold change 5.0, padj < 0.001) and *Streptococcus salivarius* (log2 fold change 5.1, padj = 0.004) were increased in follow-up compared to end of treatment.

**TABLE 2:** Alpha diversity measures between time points.

Alpha diversity measures	Pulmonary exacerbation (*n* = 34*)	End of treatment (*n* = 34*)	*P*-value	End of treatment (*n* = 29^†^)	Follow up (*n* = 29^†^)	*P*-value
Species observed (mean, SD)	21.1 (19.3)	14.8 (13.5)	0.133	14.7 (13.0)	35.6 (29.5)	<0.001
Shannon index (mean, SD)	1.244 (0.876)	1.226 (0.696)	0.920	1.236 (0.627)	1.647 (0.982)	0.047
Inverse simpson index (mean, SD)	3.125 (2.559)	2.975 (1.807)	0.745	2.861 (1.523)	4.680 (3.784)	0.020

SD, standard deviation.

^*^For the paired *t*-test, the number of comparisons is limited to the number of end of treatment samples (*n* = 33).

^†^For the paired *t*-test, the number of comparisons is limited to the number of paired samples (*n* = 27).

### 3.3. Stratified differential abundance of bacterial genes across changes in clinical status

**FIGURE 4:**
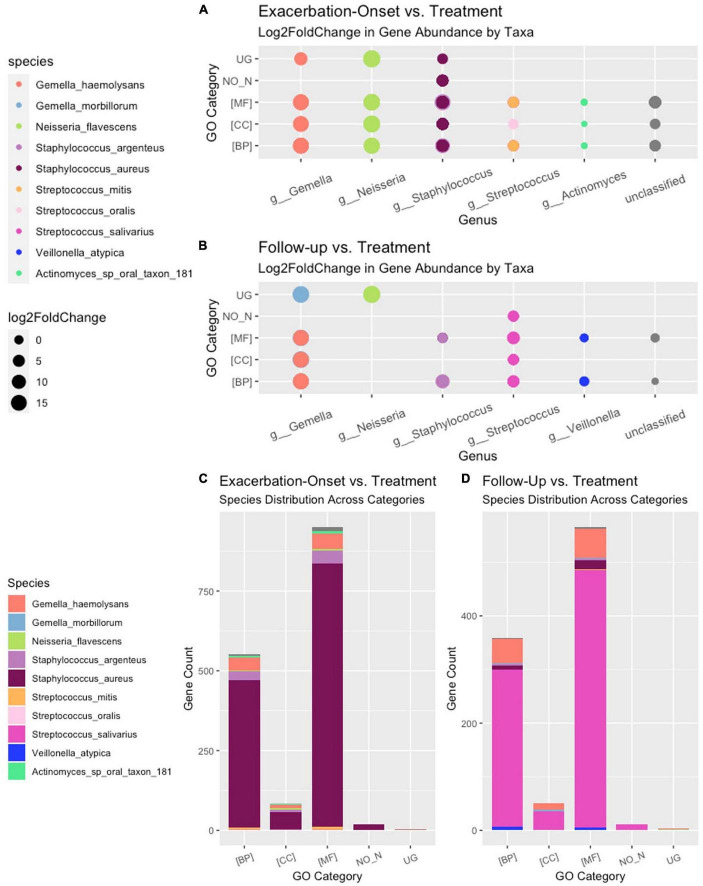
Stratified differential abundance of bacterial genes between changes in clinical status. Panel **(A)** Log2 fold change distribution across bacterial species in Follow-up compared to Treatment across GO categories. Panel **(B)** Log2 fold change distribution across bacterial species in Exacerbation Onset vs. Treatment across GO categories. Panel **(C)** Stratified differential abundance of bacterial genes in Follow-up compared to Treatment across GO categories. Panel **(D)** Stratified differential abundance of bacterial genes in Exacerbation-Onset compared to treatment across GO categories. GO, gene ontology; BP, biological processes; MF, molecular functions; CC, cellular components. The genus name is centered on each column shown.

**FIGURE 5:**
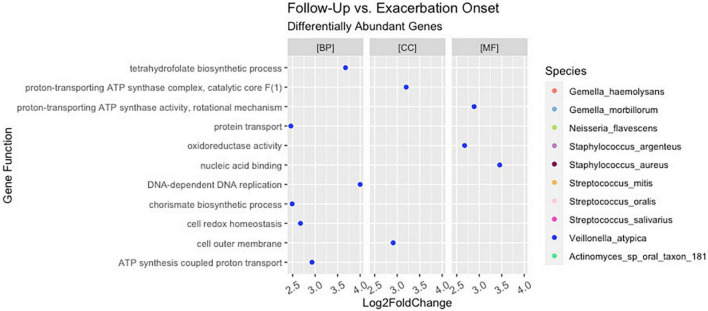
Differential abundance of *Veillonella atypica* genes in follow-up versus pulmonary exacerbation. While ten bacterial species were identified to have a differential abundance of bacterial genes across clinical states (i.e., pulmonary exacerbation, end of treatment, and follow-up), only *Veillonella atypica* had a differential abundance of bacterial genes when comparing follow-up pulmonary exacerbation onset. BP, biological processes; MF, molecular functions; CC, cellular components.

Of the 221 total bacteria identified in the dataset, a total of ten species (4.5%) displayed significant (padj < 0.01 and log2 fold change > |2|) differential gene abundance across changes in clinical status. These included *Gemella haemolysans*, *Gemella morillorum*, *Neisseria flavescens*, *Staphylococcus argenteus*, *Staphylococcus aureus*, *Streptococcus mitis*, *Streptococcus oralis*, *Streptococcus salivarius*, *Veillonella atypica*, and *Actinomyces*_sp_oral_taxon_181 (Figure 4). Interestingly, the species *Gemella morbillorum* and *Veillonella atypica* contained differentially abundant genes in follow-up compared to end of treatment but did not in PEx compared to end of treatment. Similarly, the *Actinomyces* sp. was not present in follow-up versus end of treatment (Figure 4). As shown in Figure 5, when we compared the gene abundances at follow-up and PEx, we found that each of the eleven genes found belonged to *Veillonella atypica* in follow-up.

A total of 192,714 genes were identified across all samples. Of these genes, across all comparisons, 1,673 were found to be significantly differentially abundant with padj < 0.01 and log2 fold change > |2|. When comparing the gene abundances at PEx and end of treatment, there were 1,608 significantly differentially abundant genes in PEx distributed across all three Gene Ontology (GO) categories with 552 genes in “Biological Processes” [(BP)], 951 genes in “Molecular Functions” [(MF)], 83 genes in “Cellular Components [(CC)], and 22 genes left uncategorized (Figure 4C and Table 3). When comparing the gene abundance at follow-up and end of treatment, there were 989 significantly differentially abundant genes in follow-up with 358 genes in [BP], 566 genes in [MF], 50 genes in [CC], and 15 left unidentified (Figure 4D and Table 3).

**TABLE 3:** Gene and pathway abundance of clinical status comparisons (across respective categories).

Categories	Unique exacerbation	Unique follow-up	Sig follow-up vs. exacerbation	Sig exacerbation vs. end of treatment	Sig follow-up vs. end of treatment
Genes	673	128	11	1608	989
[BP] Genes			6	552	358
[MF] Genes			2	951	566
[CC] Genes			3	83	50
Uncategorized			–	22	15
Pathways	53	13	1	106	66
Amine and polyamine degradation	1	–	–		
Amino acid biosynthesis	5	5	–		
C1 compound utilization and assimilation	2	–	–		
Carbohydrate biosynthesis	1	4	1		
Carbohydrate degradation	–	3	–		
Cell structure biosynthesis	4	1	–		
Cofactor, prosthetic group, electron carrier, and vitamin biosynthesis	18	–	–		
Fermentation	1	–	–		
Glycolysis	3	–	–		
Inorganic nutrient metabolism	1	–	–		
Nucleic acid processing	1	–	–		
Nucleoside and nucleotide biosynthesis	8	–	–		
Other biosynthesis	1	–	–		
Pentose phosphate pathway	1	–	–		
Polyprenyl biosynthesis	2	–	–		
Secondary metabolite biosynthesis	2	–	–		
Tetrapyrrole biosynthesis	2	–	–		

E, pulmonary exacerbation; F, follow-up; T, end of treatment; BP, biological processes; MF, molecular functions; CC, cellular components.

**FIGURE 6:**
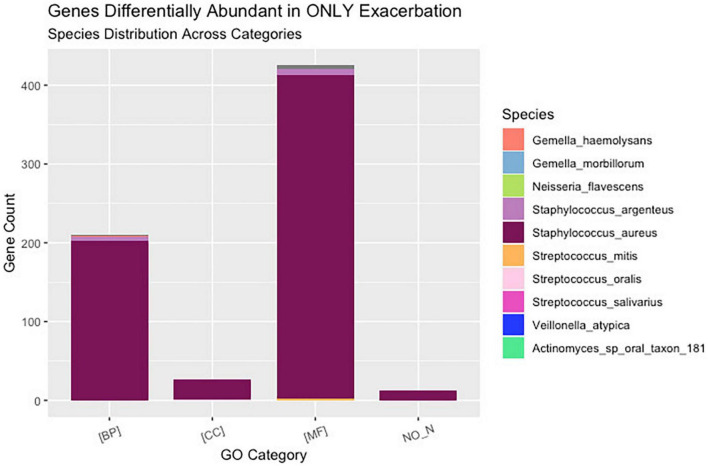
Uniquely differentially abundant genes at pulmonary exacerbation versus end of treatment. While ten bacterial species were identified to have a differential abundance of bacterial genes across clinical states (i.e., pulmonary exacerbation, end of treatment, and follow-up), the majority of bacterial genes differentially abundant in exacerbation belonged to *Staphylococcus aureus*. GO, gene ontology; BP, biological processes; MF, molecular functions; CC, cellular components.

Interestingly, although the comparisons of PEx and follow-up versus end of treatment displayed similar distributions of genes across GO categories (Figures 4C, D), the species demonstrating these changes in each comparison to treatment differed. The species *Staphylococcus aureus* accounted for eighty-one percent of the differential gene abundance in PEx with 1,306 genes represented in all three GO categories. Meanwhile, the species *Streptococcus salivarius* accounted for eighty-three percent of the differential gene abundance in follow-up with 821 genes represented in all three GO categories. Additionally, of the 989 genes differentially abundant in follow-up vs. end of treatment, 128 (121 belonging to *Streptococcus salivarius*) genes differed from those in PEx. This number was dwarfed by the 673 (651 belonging to *Staphylococcus aureus*) genes that were unique to PEx (not differentially abundant in follow-up vs. end of treatment) (Figure 6). In the comparison of follow-up to PEx, eleven total genes spread across all three GO Categories were found to be differentially abundant in follow-up, with log2 fold changes ranging from 2.5–3.5 (Figure 5).

### 3.4. Stratified differential abundance of bacterial metabolic pathways across changes in clinical status

**FIGURE 7:**
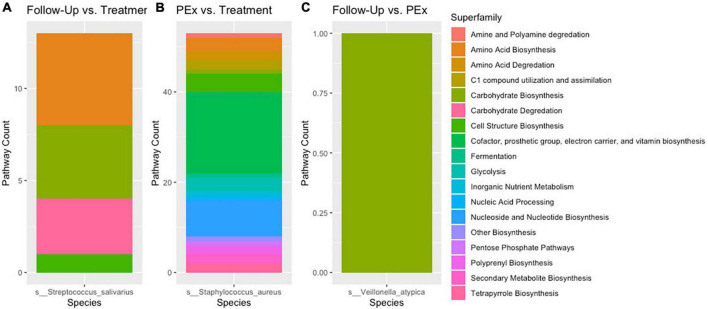
Stratified differential pathway abundance. Panel **(A)** Metabolic pathways attributed to *Streptococcus salivarius* were differentially abundant between follow-up and treatment. Panel **(B)** Metabolic pathways attributed to *Staphylococcus aureus* were differentially abundant between pulmonary exacerbation and treatment. Panel **(C)** A single metabolic pathways attributed to *Veillonella atypica* was differentially abundant between follow-up and pulmonary exacerbation. When comparing the metabolic pathways between clinical states (i.e., pulmonary exacerbation, end of treatment, and follow-up), only one bacterial species was differentially abundant between each comparison. The different metabolic pathways are shown in different colors for each bacterial species. PEx, pulmonary exacerbation.

There were 8,653 metabolic pathways identified across all samples. Of these pathways, across all comparisons, 120 were found to be significantly differentially abundant with padj > 0.05 and log2 fold change > |2|. When comparing PEx and follow-up to end of treatment, 106 and 66 pathways were found to be significantly differentially abundant, respectively. Interestingly, in the PEx versus end of treatment comparison, all 106 pathways occurred in *Staphylococcus aureus*. Likewise, in the follow-up versus end of treatment comparison, all 66 pathways occurred in *Streptococcus salivarius*. There were 53 pathways that were uniquely differentially abundant in PEx; spanning across 16 of the 17 total MetaCyc pathway superfamilies detected (Table 3). The “Cofactor, prosthetic group, electron carrier, and vitamin biosynthesis” and “Nucleotide and Nucleotide Biosynthesis” were the most substantial superfamilies, containing 18 and 8 metabolic pathways, respectively. There were 13 unique differentially abundant metabolic pathways in follow-up compared to end of treatment spanning across four MetaCyc superfamilies. “Carbohydrate Biosynthesis” contained the most pathways with 4, followed by “Carbohydrate Degradation with 3 (Figure 7). Most notably, when we compared follow-up to PEx, we found that *Veillonella atypica* species possessed the only differentially abundant metabolic pathway: UDP-N-acetyl-D-glucosamine biosynthesis (from the “Carbohydrate Biosynthesis” superfamily; see^1^ for an image of the pathway). The product of this pathway is N-acetyl-glucosamine (GlcNac), which has been shown to activate glycolysis and may affect community interactions between anaerobes and aerobes in the CF airway.

## 4. Discussion

In this study, we found that only 5% of the bacterial species observed displayed differential gene abundance between changes in clinical status across all comparisons. The most significant findings from the PEx vs. end of treatment and follow-up vs. end of treatment comparisons were the differentially abundant genes/pathways of *Staphylococcus aureus* in PEx and those of *Streptococcus salivarius* in follow-up. When comparing to end-of treatment, PEx and follow-up displayed similar differential gene abundance with genus’ such as *Gemella* sp., *Neisseria* sp., and other *Streptococcus* species. As various broad-spectrum antibiotics such as those administered to our patient dataset have commonly been known to target many bacterial species and decrease their metabolic activity, we attributed these similarities to being an effect of antibiotic treatment and therefore inconsequential to the onset of PEx. Additionally, although PEx and follow-up differed in genus’ such as *Veillonella* sp. and *Actinomyces* sp., the gene abundances made up such a small portion of the differentially abundant genes that they were also ruled as inconsequential. Perhaps most intriguingly, in the comparison of follow-up and PEx, we found that the only species with differentially abundant genes in follow-up was the anaerobic species of *Veillonella atypica*. The follow-up vs. PEx comparison additionally only yielded one differentially abundant pathway: the UDP-N-acetyl-D-glucosamine biosynthesis I pathway in *Veillonella atypica*.

Our results suggest that *Veillonella atypica* and N-acetylglucosamine may play a potential role in improved lung function. The anaerobic bacteria most universally found in CF patient’s lungs are *Veillonella* and *Prevotella* (common oral commensals) (26–28). While the role of anaerobes in the CF airway still remains unclear (29), orally derived anaerobic bacteria occupy distinct niches distinct from *Staphylococcus* or *Pseudomonas* and the naturally anoxic conditions actually favor anaerobic growth that may contribute to altered ecosystem dynamics (7, 30–32). Studies have shown occurrences of both positive and negative correlations between the presence of anaerobic bacteria and lung function in CF patients (27–29, 32), however, studies focusing on *Veillonella* itself have reported a positive correlation with the presence of this anaerobe and milder disease in patients with CF (28, 33). In our study, we found that *Veillonella* was only differentially abundant compared to the PEx clinical state but not compared to the end of treatment state (when the patient is no longer experiencing a sharp decrease in lung function). Given its positive associations with milder disease as well as its displayed “absence” in the PEx clinical state, we surmised that the metabolic activity of *Veillonella atypica* may play an influential role in the CF lung microbiome.

The only differentially abundant pathway in follow-up vs. PEx was the UDP-N-acetyl-D-glucosamine biosynthesis I pathway in *Veillonella atypica*. The product of this pathway is N-acetyl-glucosamine (GlcNac), which can serve as a precursor for peptidoglycan or function in glycolysis by catabolizing to fructose 6-phosphate (Fru6P) (34, 35). Studies have shown that antibiotic tolerance in bacteria can be greatly enhanced when the bacteria are deprived of essential nutrients (36, 37). A prior study found that the presence of GlcNac not only activated glycolysis in *Escherichia coli*, but it also reactivated the *Escherichia coli*’s peptidoglycan biosynthesis process (38). The abundance of the UDP-N-acetyl-D-glucosamine biosynthesis I pathway in follow-up (and lack thereof in PEx) may point to important community interactions between anaerobes and aerobes which may influence the onset of the PEx clinical state. This finding may also provide context to why the abundance of *Veillonella* has been positively correlated to increased lung function in patients with CF in past studies.

Our data also supports that *Staphylococcus aureus* adapts to a nutrient-limited CF lung environment. The majority of the differentially abundant genes in follow-up compared to end of treatment belonged to *Streptococcus salivarius* and those in PEx belonged to *Staphylococcus aureus*. Moreover, when the genes uniquely abundant at PEx compared to end of treatment (not present in follow-up vs. treatment) were sequestered, we found that the vast majority belonged to *Staphylococcus aureus*. Additionally, *Staphylococcus aureus* was the only species with differentially abundant functional profiles in PEx vs. end of treatment and displayed the largest number of metabolic pathways and MetaCyc superfamilies across all three comparisons. It is reasonable to surmise that the 673 genes uniquely abundant in *Staphylococcus aureus* during PEx (before antibiotic treatment), but not during follow-up (after antibiotic treatment) were associated with the decrease in lung function (PEx) that the persons with CF experienced. *Staphylococcus aureus* is the most common pathogen isolated from CF persons and has strong associations with inflammatory activity in the lung (39, 40). This species also has a large genetic diversity in persons with CF in comparison those isolated from the lungs of healthy individuals (39, 41). A compelling argument for *Staphylococcus aureus*’s association with PEx is its reputation for promoting pathogenic genes. Studies have shown *Staphylococcus aureus* to promote genes associated with persistence during chronic infection and antimicrobial resistance during antibiotic treatment (39, 42). *Staphylococcus aureus* has also displayed participation in certain adapted bacterial community interactions with *Pseudomonas aeruginosa* (43, 44), which have been strongly correlated with chronic respiratory diseases.

A consequence of the chronic PEx events that persons with CF experience due to chronic infection is the associated inflammation and subsequent tissue damage which leads to remodeling of the epithelial cell architecture (26, 45). This remodeling, combined with the anoxic and acidic conditions characteristic to the CF lung, poses conceivable selective pressures on the lung microbiome (26, 44). As expected, in past studies, *Staphylococcus aureus* was found competing with *Pseudomonas aeruginosa* for limited resources when in the CF lung environment (7, 44). In prior studies of *Escherichia coli*, it was found that inactivating the nucleotide biosynthesis genes significantly decreased the *Escherichia coli*’s ability to produce the enzymes necessary for metabolite biosynthesis (46). Genes involved in nucleotide metabolism were also required for *Pseudomonas aeruginosa* survival in an *in vivo* and an *in vivo*-like model (47). This aligns with the finding that the largest number of differentially abundant metabolic pathways in PEx vs. end of treatment belonged to the “cofactor, prosthetic group, electron carrier, and vitamin biosynthesis” and “nucleotide and nucleoside biosynthesis” pathways of *Staphylococcus aureus*. The gene and path abundance findings in our study also suggest that *Staphylococcus aureus* is not only capable of metabolite and nucleotide biosynthesis, but that this ability may contribute to the chronic infection and inflammation that persons with CF experience.

The role of *Streptococcus* in the lung is lesser known, but the genus has been growing in recognition as a central component of the CF lung microbiome with a highly variable genome (48, 49). The differential gene abundance in follow-up versus end of treatment, as well as all 128 uniquely expressed genes (not in PEx vs. end of treatment) belonging to *Streptococcus salivarius* have the potential to have contributed negatively and/or positively. While it has been linked in some studies to PEx (when it was the predominant species in the CF lung), its higher relative abundance has also correlated with less severe lung disease than other pathogenic species known to inhabit the CF lung microbiome (48).

There are a few limitations to note with this study. First, it was a single center study and limited to children and young adults who could spontaneously produce sputum at PEx onset. Thus, the total number of study participants is small and skews toward those with more symptomatic disease, which may impact the generalizability of these results. Second, the data was collected around a single PEx and not longitudinal in nature. A longer longitudinal study could have provided more insight into these metabolic markers as predictors for future PEx events.

In summary, around 5% of bacteria species observed had a differential gene abundance across clinical states. Three of these species, *Staphylococcus aureus*, *Streptococcus salivarius*, and *Veillonella atypica*, accounted for the most change. Further, several of the metabolic pathways identified in our dataset have been previously shown to affect lung function and lung inflammation. Taken together, these data suggest the metabolic potential of bacterial species can provide more insight into changes across clinical states than the relative abundance of bacteria alone.

## Data availability statement

The datasets presented in this study can be found in online repositories. The names of the repository/repositories and accession number(s) can be found below: https://www.ncbi.nlm.nih.gov/, PRJNA825831.

## Ethics statement

The studies involving human participants were reviewed and approved by Institutional Review Board, Children’s National Hospital. Written informed consent to participate in this study was provided by the participants’ legal guardian/next of kin.

## Author contributions

AH conceptualized and designed the study with support from AK, KC, and EZ. AH, AB, HC, IS, and AK were all involved in study participant recruitment. AH and AB performed data collection. AB performed sample processing and DNA extraction. KC supervised metagenomic sequencing. GS performed bioinformatic analysis and data analysis and generated figures, with assistance from AH. AH, RF, KC, and EZ were involved in selection of analysis methods and interpretation of findings. GS wrote the original manuscript. All authors were involved in manuscript revision and approved of the final version as written.

## Footnotes

1
https://biocyc.org/META/NEW-IMAGE?type=PATHWAY&object=UDPNAGSYN-PWY
